# Progranulin Deficient Mice Develop Nephrogenic Diabetes Insipidus

**DOI:** 10.14336/AD.2017.1127

**Published:** 2018-10-01

**Authors:** Stefanie Hardt, Lucie Valek, Jinyang Zeng-Brouwers, Annett Wilken-Schmitz, Liliana Schaefer, Irmgard Tegeder

**Affiliations:** ^1^Clinical Pharmacology, Goethe-University Hospital Frankfurt am Main, Germany; ^2^General Pharmacology and Toxicology, Goethe-University Hospital Frankfurt am Main, Germany

**Keywords:** Progranulin, aging, polyuria, polydipsia, knockout mice, hypothalamus, vasopressin

## Abstract

Loss-of-function mutations of progranulin are associated with frontotemporal dementia in humans, and its deficiency in mice is a model for this disease but with normal life expectancy and mild cognitive decline on aging. The present study shows that aging progranulin deficient mice develop progressive polydipsia and polyuria under standard housing conditions starting at middle age (6-9 months). They showed high water licking behavior and doubling of the normal daily drinking volume, associated with increased daily urine output and a decrease of urine osmolality, all maintained during water restriction. Creatinine clearance, urine urea, urine albumin and glucose were normal. Hence, there were no signs of osmotic diuresis or overt renal disease, other than a concentrating defect. In line, the kidney morphology and histology revealed a 50% increase of the kidney weight, kidney enlargement, mild infiltrations of the medulla with pro-inflammatory cells, widening of tubules but no overt signs of a glomerular or tubular pathology. Plasma vasopressin levels were on average about 3-fold higher than normal levels, suggesting that the water loss resulted from unresponsiveness of the collecting tubules towards vasopressin, and indeed aquaporin-2 immunofluorescence in collecting tubules was diminished, whereas renal and hypothalamic vasopressin were increased, the latter in spite of substantial astrogliosis in the hypothalamus. The data suggest that progranulin deficiency causes nephrogenic diabetes insipidus in mice during aging. Possibly, polydipsia in affected patients - eventually interpreted as psychogenic polydipsia - may point to a similar concentrating defect.

Progranulin is a multi-functional secreted neuroprotective and immune-regulatory protein. Loss-of-function mutations in humans are associated with ubiquitin positive, tau-negative frontotemporal lobar degeneration (FTLD) [1, 2], lipofuscinosis [3] and other rare neurodegenerative diseases [4]. Its deficiency in mice partly mimics the human disease, in particular the neuropsychiatric behavioral abnormalities [5], which are characteristic for FTLD patients whereas learning and memory are only mildly impaired [6-8]. Progranulin is expressed in neurons of the peripheral and central nervous system, but also by activated immune cells including macrophages and microglia, but knockout models suggest that the neurodegeneration is rather the origin than sequela of the neuro-inflammation [9]. It is still under debate whether progranulin released by immune cells provides a usable reservoir for neurons, either by being internalized via a transporter like sortilin [10] or via receptor-mediated endocytosis and signaling via EGF receptors [11], Notch receptors [12] and Ephrin A2 [13], all recognized as progranulin receptors. Sortilin is likely dispensable for progranulin’s neurotrophic effects [14] but in the context of the kidney, it is noteworthy that progranulin shares sortilin as transporter with the sphingolipid activating protein, prosaposin [15, 16], a precursor of saposins, whose deficiency results in renal pathology [17] and neurodegeneration [18] due to insufficient degradation of glycosphingolipids [17, 19].

Progranulin’s functions in the periphery are still somewhat enigmatic. It promotes wound healing of the skin [20], reduces joint inflammation in arthritis [21], protects the kidney against ischemia-reperfusion injury [22] and promotes tumor growth [11], all supposed to result from silencing of an activated immune system [21]. Intracellular progranulin is localized to vesicular structures, which are endosomes or autophagolysosomes and it likely promotes the degradation of protein and lipid waste via the respective pathways [23, 24]. Gene ontology enrichment analyses suggest that it contributes to the regulation of vesicular transport of proteins and metals [23]. In particular, its deficiency is associated with dysregulations of zinc transporter [5] including Slc30a9, which has been recently associated with a rare cortico-renal disease in humans [25].

We have previously observed that aged progranulin deficient mice, despite their learning deficits, make few errors in place preference learning tasks, if water was the provided award [8], suggesting a higher appetitive drive Indeed, under standard housing conditions, they drank more water, which became apparent at 12-15 months of age Considering its multiple functions for neuronal and peripheral-immune homeostasis, polyuria might be due to primary polydipsia like in saposin D deficient mice [26], hence reflecting FTLD-like impulsive drinking and feeding The polydipsia may also result from neurodegeneration in hypothalamus or pituitary gland, resulting in alterations of vasopressin production, supported by a previous study showing feeding-dependent fluctuations of progranulin release in the hypothalamus [27] Progranulin deficiency also impairs the development of sexual dimorphisms [28], suggesting that hormone-producing neurons are particularly vulnerable Finally, the polydipsia may be caused by osmotic glucosuria or a manifestation of a nephrogenic diabetes insipidus, the latter due to unresponsiveness towards vasopressin all resulting in renal water losses Progranulin indeed regulates insulin sensitivity [29, 30], possibly by interfering with glucose transporters of the solute carrier family [31], is involved in vesicular transport [23] and glycogen synthase kinase functions [32], which are essential mechanisms for the insertion of water channels into membranes [33]

To dissect out the underlying cause, which may be clinically relevant for progranulin-deficient patients, we assessed drinking and feeding behavior, metabolic functions, plasma vasopressin and other neuropeptides, morphology of the kidney and hypothalamus and aquaporins in aged progranulin deficient and control mice, and in summary, the data reveal a renal water loss without glucosuria with high vasopressin suggesting a nephrogenic diabetes insipidus.

## MATERIALS AND METHODS

### Animals

Progranulin knockout mice (Grn^-/-^) [34] were maintained as homozygous colony. The background is C57BL6J, so that sex and age matched C57BL6J mice (Charles River or Envigo, Germany) were used as controls. Mice were housed three to five per cage and maintained in the same room during life with constant room temperature (21 ± 1°C), standard diet and a regular light/dark schedule with light on from 7:00 A.M. to 7:00 P.M. Food and water was available ad libitum except for some experimental sessions in the IntelliCage and metabolic cage. Young mice were 9-12 weeks, aged mice 10-13 months and old mice 15-18 months. The experiments were approved by the local Ethics Committee for Animal Research (Darmstadt, Germany) and adhered to the guidelines of GV-SOLAS for animal welfare in science and the ARRIVE guidelines.

### IntelliCage Licking behavior

The Intellicage (NewBehavior AG, Zurich, Switzerland) [8, 35] consists of four operant corners, each with two water bottles, sensors, light-emitting-diodes (LEDs) and doors that control the access to the water bottles. The system fits into a large cage (20 x 55 x 38 cm, Tecniplast, 2000P). Four triangular red shelters (Tecniplast) are placed in the middle to serve as sleeping quarters and as stands to reach the food. The floor is covered with thick bedding. Mice are tagged with radio-frequency identification (RFID)-transponders, which are read with an RFID antenna integrated at corner entrance. Inside the corners, there are two holes with water bottles, which can be opened and closed by automated doors. Mice have to make nosepokes (NP) to open the doors for water access. The numbers and duration of corner visits, nosepokes, and licks are automatically recorded without the need for any handling of the mice during the recording times. Sixteen mice were housed in each cage.

The IntelliCage experiments followed established protocols [8, 35, 36]. Mice were adapted to the system for 3 days with free access to every corner, with all doors open, water and food ad libitum. The free adaptation was followed by 4-days "nosepoke adaptation", during which the doors upon nosepoking at the door. Water was available for 24h. Mice were then adapted for 7 days to the "drinking-session" protocol, in which drinking was allowed between 11 and 13 a.m. and 4 and 6 p.m. Outside of these times the doors remained closed. The day-pattern was used to increase the motivation to learn. The restricted drinking times were maintained in subsequent learning tasks. Subsequently, mice returned to their home cages with free access to food and water.

### Phenomaster

The TSE Phenomaster offers an automated metabolic and behavioral monitoring in home cage environments. Drinking and feeding behavior were monitored with high-precision weight sensors for liquid and food dispensers, which are integrated into the lid of the cage. Mice were adapted to the drinking bottles for one week in their home cage and to the Phenomaster® cage for 3 consecutive days before starting the experiment, which consisted in 24h tap water and 24h sweetened water (20% sucrose). Drinking and feeding were recorded for 24 hours.

### Excretion of creatinine, urea, glucose and albumin

Mice were placed in metabolic cages (Tecniplast) for urine collection with free access to water for 24h followed by water restriction for 2 x 12h with 1h free drinking in between. Urine was collected for 24 h in a cooling device. Body weight and drinking volume were determined daily. The 24h urine volume, urine specific weight, urine concentrations of creatinine, urea and glucose were analyzed by the veterinary laboratory LaboKlin, Bad Kissingen, Germany. Urinary albumin excretion was determined by the Bradford method and microalbumin test stripes (DIMA, Product ID M04S10-25). In addition, we used an ELISA for mouse albumin. Urinary creatinine levels were also assessed with the creatinine assay kit (Labor-Technik, Berlin, Germany) according to the manufacturer’s instruction.

### Serum and plasma analyses

Serum creatinine was determined with a colorimetric Microplate Assay (Oxford Biomedical Research, Biotrend, Germany), and serum urea was analyzed with an Urea Assay Kit (BioCat, Heidelberg, Germany) following the manufacturer’s instruction. Serum C-reactive protein, glucose and plasma zinc levels were analyzed by LaboKlin.

### Analysis of vasopressin and other neuropeptides

Plasma levels of vasopressin were measured by a specific enzyme immunoassays (Arg8-vasopressin EIA kit) from Enzo (Cat.No ADI-900-017, Enzo Life science GmbH, Germany) according to the instructions of the supplier. In addition, we analyzed concentration of agouti related protein (AGRP, Cat.No LS-F16813, Life Span Bioscience, USA), visinin-like protein 1 (VILIP, Cat.No CSB-EL025933MO, CusaBio Biotech, USA) and ghrelin (Cat.No EZRGRT-91K, Merck Millipore, Germany) in plasma samples using specific ELISA kits, all according to instructions of the suppliers. Vasopressin in the kidney and hypothalamus was analyzed per immune-histochemistry.

### Morphological studies and immunohistochemistry

Mice were terminally anaesthetized with isoflurane and cardially perfused with cold 1x phosphate buffered saline (PBS), pH 7.4 followed by 4% paraformaldehyde (PFA) in PBS for fixation. The kidneys were subsequently postfixed with 4% PFA in 1xPBS solution for 2h, dehydrated and embedded in paraffin. Renal sections (3.5 µm) were deparaffinized in xylene and graded ethanol and stained with haematoxylin & eosin (H&E) and periodic acid Schiff reagent (PAS). The sections were analyzed for signs of glomerulonephritis, sclerosis or tubulopathy as reported previously [37] by an investigator blinded to the genotypes. In addition, sections were processed for analysis of immune cell infiltration and aquaporin expression using immunohistology. Antigen retrieval in citrate buffer and antibody stainings followed standard procedures. Primary antibodies included rat anti-mouse F4/80 (Serotec), AQP1, AQP2 and AQP4 (Alamone labs, Jerusalem, Israel; diluted 1:100 in 1 % BSA in PBS-Triton) and vasopressin (Serotec). The numbers of neutrophils, macrophages and T cells were estimated per field (high-power field 400x, with a minimum of 10 fields counted per mouse, 3 mice per group; Soft Imaging System, Olympus, Hamburg, Germany).

For analysis of vasopressin positive neurons in the hypothalamus brains were excised, postfixed in 4 % PFA (2 h, RT), cryoprotected in 20 % sucrose (overnight, 4 °C), embedded in tissue molds in cryomedium and stored at -80 °C. Cryosections of 12 µm were permeabilized with 0.1% Triton-X-100 in 1x PBS (PBST) for 25 min, then blocked for 30 min with 5% BSA (Roth) in 1x PBS. Subsequently, sections were incubated overnight with the respective primary antibody at 4 °C, and subsequent secondary antibody labelled with Alexa-488 or Cy3 (2h, RT), followed by 30 min incubation with DAPI (1 µg/µl in 1 % BSA in PBST). Primary antibodies included vasopressin (1:500, 48h, Serotec) and GFAP (1:500, Millipore). Fluorescent sections were imbedded in Fluoromount (eBioscience). Images were captured with a Keyence (BZ-9000, Germany) microscope. For overviews, tiled images were automatically stitched. An observer who was not aware of the genotypes performed the image analyses with FIJI ImageJ for quantification of aquaporin and vasopressin immunofluorescence. The Particle Counter was employed after setting of the threshold (Isodata algorithm) and definition of size inclusion and circularity criteria. The analysis was done with sections of 4 mice per group. Results are presented as number or particles or percentage of area coverage with positive particles.

### Statistics

Data are presented as mean ± SD unless stated otherwise and were analyzed with SPSS 23 and Graphpad Prism 6.0. Groups comprised 6-16 mice depending on the experiment, and numbers are presented in the figures and/or figure legends Data were submitted to univariate analysis of variance (ANOVA) or unpaired, 2-tailed Student’s t-tests. In case of significant differences of ANOVAs, groups were compared with the respective control groups using t-tests with a correction of alpha according to Dunnett (one control group) or according to Šidák (specific control groups). Wildtype young mice were mostly used as the reference group. The alpha level was set to 0.05 for all comparisons, and adjusted P-values are reported.

**Figure 1.**
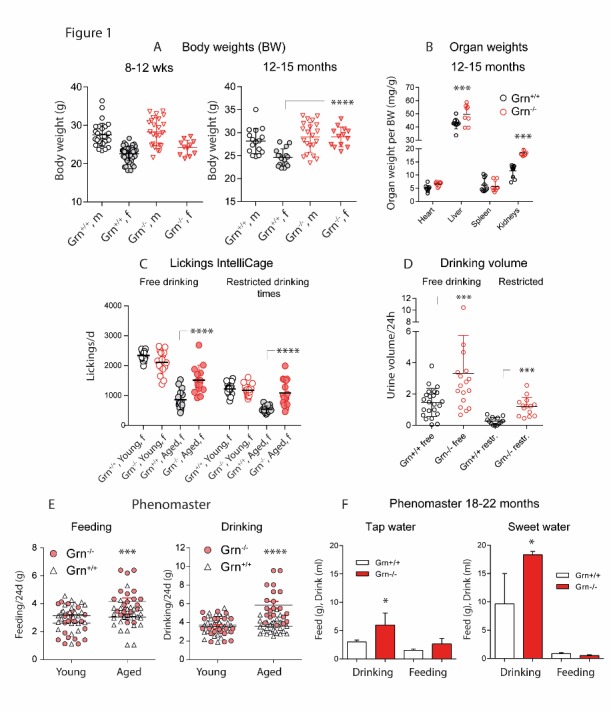
Body weight, drinking and feeding behavior of progranulin deficient (Grn^-/-^) and control mice (Grn^+/+^) **A)** Scatter plots showing the body weight of young and aged male and female Grn^-/-^ and Grn^+/+^ mice. **B)** Organ weights of aged Grn^-/-^ and Grn^+/+^ mice. **C)** Number of lickings within 24h in the IntelliCage during free-drinking and restricted-drinking experiments. During ’free-drinking’, access to the water bottles was allowed for 24h on nosepoking at the doors. During ’restricted-drinking’, access to the water bottles was mostly denied except for 2x2h per day (11-12 am and 4-5 pm). **D)** Scatter plots showing the 24h drinking volume of aged Grn^-/-^ and Grn^+/+^ mice during free drinking and water restriction for 2x12h with one-hour free drinking in between. **E)** Scatter plots showing the weight of food and water consumed within 24h in sex-matched young and aged Grn^-/-^ and Grn^+/+^ mice in the Phenomaster cage. **F)** Phenomaster analysis of drinking and feeding behavior of old Grn^-/-^ and Grn^+/+^ mice during presentation of tap water or sweet water with 20% sucrose (mean ± SD). The data show the food weight and drinking volume consumed within 24h. For all subpanels each scatter represents one mouse, the line is the mean and the whisker show the standard deviation (SD). Asterisks indicate statistically significant differences between genotypes (unpaired, 2-tailed Student’s t-test for each gender, organ, drinking or feeding. * P< 0.05, ** P<0.01, *** P<0.001, **** P<0.0001).

## RESULTS

### Body weight, feeding and drinking behavior

Under standard housing conditions, body weight was similar in male and female young Grn^+/+^ and Grn^-/-^ mice (Fig. 1A) but aged (12-15 months) female Grn^-/-^ mice were overweight (ANOVA F(3, 62) = 10.40; P < 0.0001, posthoc adjusted P-values in the figure), which was lost again at old age (>18 months, not shown). Organ sizes and weights of the heart and spleen were similar in both genotypes irrespective of the age, but the kidneys of old Grn^-/-^ mice were enlarged with increased weights (2-way ANOVA for ’group X organ’ F(3, 72) = 4.435; P = 0.0064; Fig. 1B), which was also obvious in tissue sections (Fig. 4). Significance was maintained when kidney weights were corrected for body weights, which also revealed higher relative weights of the livers (ANOVA F(3, 48) = 4.906; P = 0.0047; posthoc corrected P-values in the figure).

**Figure 2.**
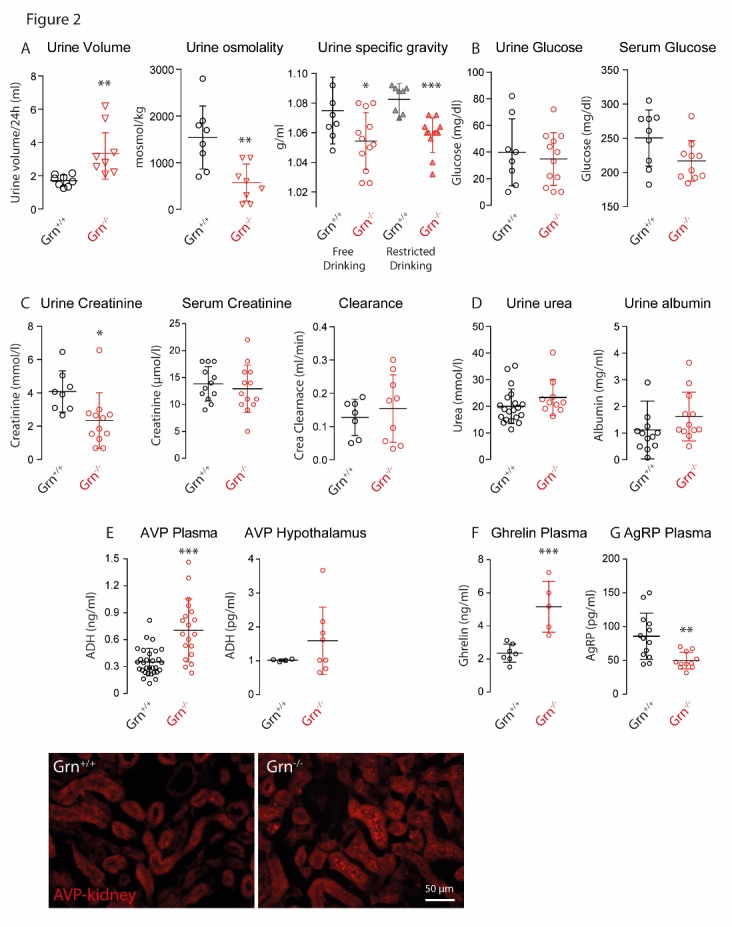
Renal and metabolic functions of progranulin deficient (Grn^-/-^) and control mice (Grn^+/+^) **A)** Scatter plots showing the 24h urine volume, urine osmolality and urine specific gravity of aged Grn^-/-^ and Grn^+/+^ mice (12-16 months old). **B)** Concentration of glucose in 24h-urine and plasma of aged Grn^-/-^ and Grn^+/+^ mice. **C)** Creatinine concentrations in 24h-urine and plasma, and creatinine clearance of aged Grn^-/-^ and Grn^+/+^ mice. **D)** Concentrations of urea and albumin in 24h-urine of aged Grn^-/-^ and Grn^+/+^ mice. **E)** Concentrations of arginine vasopressin (AVP, antidiuretic hormone) in plasma and in crude tissue extracts of the hypothalamus of aged Grn^-/-^ and Grn^+/+^ mice and immunofluorescence analysis of AVP in the kidney (bottom, scale bar 50 µm). **F, G)** Plasma concentrations of ghrelin and agouti related protein (Agrp) of aged Grn^-/-^ and Grn^+/+^ mice. For all subpanels each scatter represents one mouse, sexes were matched between groups, the line is the mean and the whisker show the standard deviation (SD). Asterisks indicate statistically significant differences between genotypes (unpaired, 2-tailed Student’s t-test. * P< 0.05, ** P<0.01, *** P<0.001).

**Figure 3.**
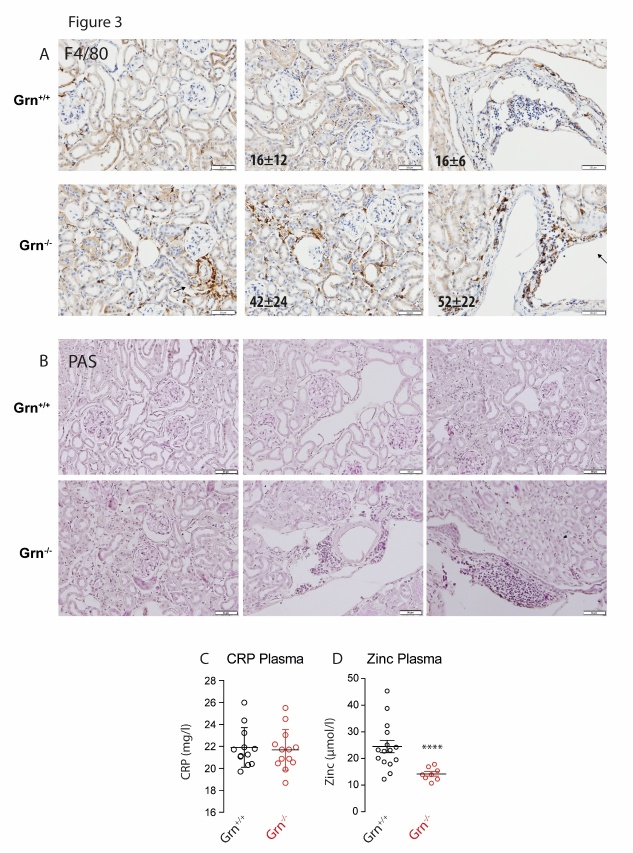
Histomorphology of the kidney of aged progranulin deficient (Grn^-/-^) and control mice (Grn^+/+^) **A)** Immunostaining of myeloid-derived F48/80-positive immune cells (brown), with hematoxylin counterstaining of nuclei (blue). Immune cells were counted per field of view and averaged (10 fields per mouse of 3 mice per group). Numbers differed significantly between genotypes (unpaired, 2-tailed Student’s t-test). Scale bars 50 µm. **B)** Periodic acid-Schiff (PAS) staining of polysaccharides and mucous substances. Histomorphometric scores did not differ between genotypes, except for a higher number of immune cells in Grn^-/-^ mice. Scale bars 50 µm. **C, D)** Concentrations of C-reactive protein (CRP) and zinc in plasma of aged Grn^-/-^ and Grn^+/+^ mice. Asterisks indicate statistically significant differences between genotypes (unpaired, 2-tailed Student’s t-test, **** P<0.0001).

**Figure 4.**
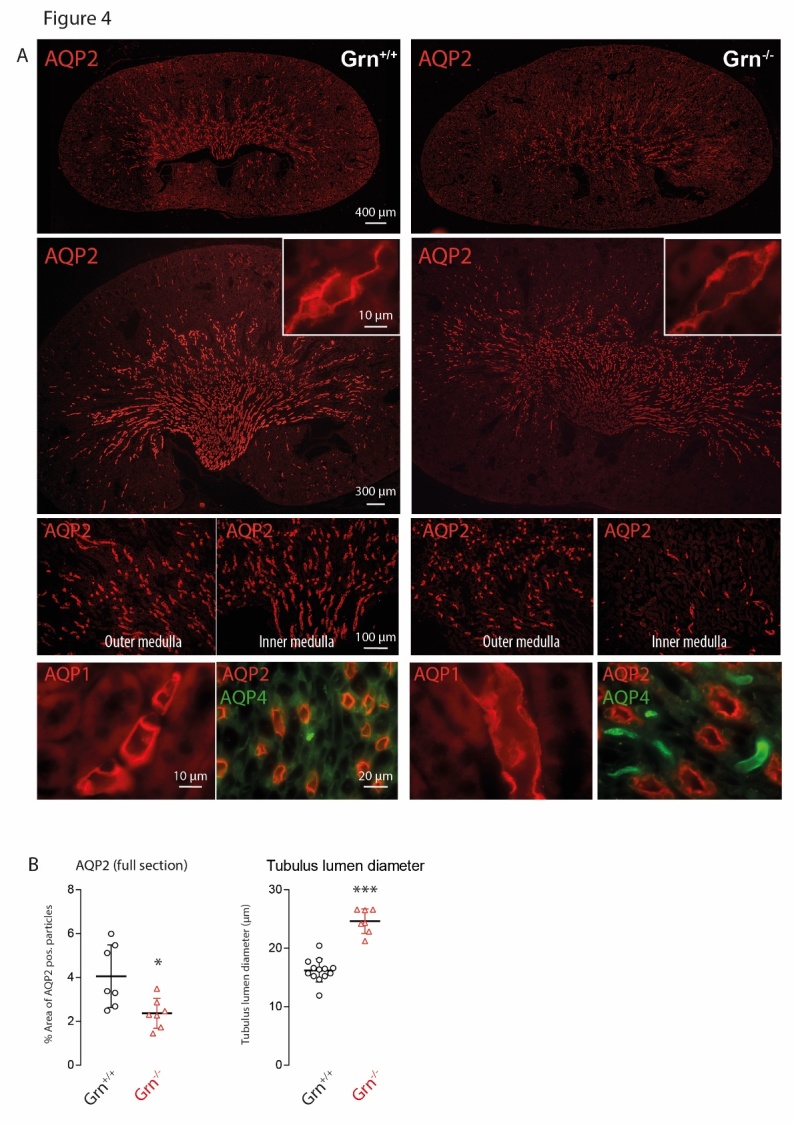
Immunofluorescence analysis of aquaporin 1, 2 and 4 (AQP1, AQP2, AQP4) in the kidney of aged progranulin deficient (Grn^-/-^) and control mice (Grn^+/+^) **A)** Examples of AQP2 immunofluorescence at low (upper panels) and high magnifications (bottom panels and insert). Scale bars as indicated in the figure. The bottom panel also shows high AQP1 and AQP4 at high magnification, which did not differ between genotypes. **B)** Quantification of AQP2 positive particles using stitched full sections of the kidney of 4 mice per group. Each scatter is a section. Analysis of the tubule lumen as assessed by measuring the lumen area in 4 sections of 4 mice per group. Each scatter is one AQP2 positive collecting duct. The images suggest reduced AQP2 expression and widening of AQP2+ collecting ducts. Asterisks indicate significant differences between genotypes, unpaired 2-tailed Student’s t-test *P<0.05, ***P<0.001).

Daily lickings in IntelliCage experiments of young and aged Grn^-/-^ mice and respective controls showed that the number of licks was significantly increased in aged Grn^-/-^ mice during both ‘free drinking’ and ‘restricted drinking’ as compared to controls (ANOVA for ’group’ F (3, 120) = 73.28; P < 0.0001; results of posthoc t-tests presented in Fig. 1C). In contrast, young mice behaved normally. Metabolic Cage experiments showed that polydipsia of aged Grn^-/-^ mice was associated with polyuria (Figure 1D), which was maintained during water restriction (2x12h with 1h free drinking in between).

We performed Phenomaster experiments (24h observation time) to quantify water and food consumption precisely. Drinking and feeding behavior was normal in young Grn^-/-^ mice, but increased in aged Grn^-/-^ mice as compared to the respective controls (ANOVA for ’age X genotype’ for drinking F (1, 94) = 28.28; P < 0.0001, for feeding F (1, 86) = 16.45; P = 0.0001; posthoc in Fig. 1E). Polydipsia of Grn^-/-^ was obvious at old age irrespective of tap water or sweetened water was presented, amounting to daily drinking volumes of about 7 and 18 ml/d in Grn^-/-^ mice for tap and sweet water as compared to 3 and 10 ml/d in controls (Fig. 1E). Tap water polydipsia was accompanied with overfeeding, whereas feeding dropped to a minimum in both genotypes when sweetened water was presented (Fig. 1F).

### Renal and metabolic functions

The increased drinking behavior was confirmed in an independent experiment in Metabolic Cages in a second set of aged Grn^-/-^ and Grn^+/+^ mice. Again, the 24h-drinking volume was almost doubled and was associated with an increase of the daily urine volume and a decrease of the urine osmolality and specific urine gravity, which was maintained during water restriction suggesting a renal water loss (Fig. 2A, results of unpaired, 2-sided t-tests in the figure). Urine and plasma glucose concentrations were in the normal range (Fig. 2B), hence excluding osmotic polyuria. Plasma creatinine and creatinine clearance (Fig. 2C), urea excretion and urine albumin (Fig. 2D) were all in the normal range, suggesting an isolated concentration defect without major glomerular or tubular dysfunctions due to a renal diabetes insipidus, which is normally associated with an increase of vasopressin release. Indeed, plasma vasopressin levels in progranulin deficient mice were on average about 3-fold higher than normal levels (Fig. 2E), associated with increased vasopressin levels in the hypothalamus and in the kidney, as revealed by immunofluorescence analyses. Hence, the hypothalamic counter-regulatory loop appeared to be intact, allowing for exclusion of psychogenic polydipsia, which would suppress vasopressin production, and excluding a hypophyseal secretory defect, which would also result in low plasma vasopressin levels. But alterations of feeding-regulating hormones, ghrelin and AgRP (Fig. 2F, G), which were oppositely regulated, suggest that progranulin deficiency may cause hypothalamic hormonal dys-balances, which may account for the increase of appetite.

### Kidney morphology and aquaporin water channels

The kidney function tests suggested that the renal water loss was caused by a defect of water reabsorption in tubules or collecting ducts. The histomorphology of the kidneys showed mildly widened tubules but no overt tubulopathy (Fig. 3A, B). The kidneys of aged Grn^-/-^ mice showed significantly higher numbers of F4/80+ macrophages within the interstitium and perivascular regions (Fig. 3A; renal cortex P 0.039; renal medulla P = 0.0031), but H&E and PAS stainings (Fig. 3B) did not reveal signs of glomerulonephritis, tubulonephritis or tubulosclerosis. Overall, the kidney morphology agreed with the higher urine turnover [38], possibly owing to a vasopressin un-responsiveness, but without structural changes or diseases of the kidney that may result in renal water losses such as amyloidosis. Immune cell infiltration of the kidneys was not associated with higher plasma levels of the pro-inflammation markers, C-reactive protein (Fig. 3C) and euhydrated Grn^-/-^ mice had no derangement of serum electrolytes or serum osmolality (Table 1). Urine creatinine, potassium and magnesium were reduced likely owing to secretion of diluted urine (Table 1), and levels of the trace element, zinc were reduced is plasma (Fig. 3D) confirming our previous studies [5].

Congenital nephrogenic diabetes insipidus in humans is mostly caused by loss-of-function mutations of the renal vasopressin receptor, which is required for insertion of aquaporin water channels into the outer membrane of collecting duct cells [39], or more rarely the defect is caused by mutations of aquaporin 2 [40]. To address these potential mechanisms, we performed immunofluorescence studies of aquaporin 1, 2 and 4 (AQP1, AQP2, AQP4) in the kidneys (Fig. 4). AQP1 and AQP4 immunoreactivity were similar in both genotypes. However, AQP2 immunofluorescence clearly shows the enlargement of the Grn^-/-^ kidney and widening of the tubules associated with an apparent reduction of AQP2 immunofluorescence intensity, particularly in the inner medulla. High magnification shows that AQP2 is inserted in the apical membrane but somewhat weaker than in controls, suggesting that an AQP2 deficit in the collecting tubules may account for the water loss.

**Figure 5.**
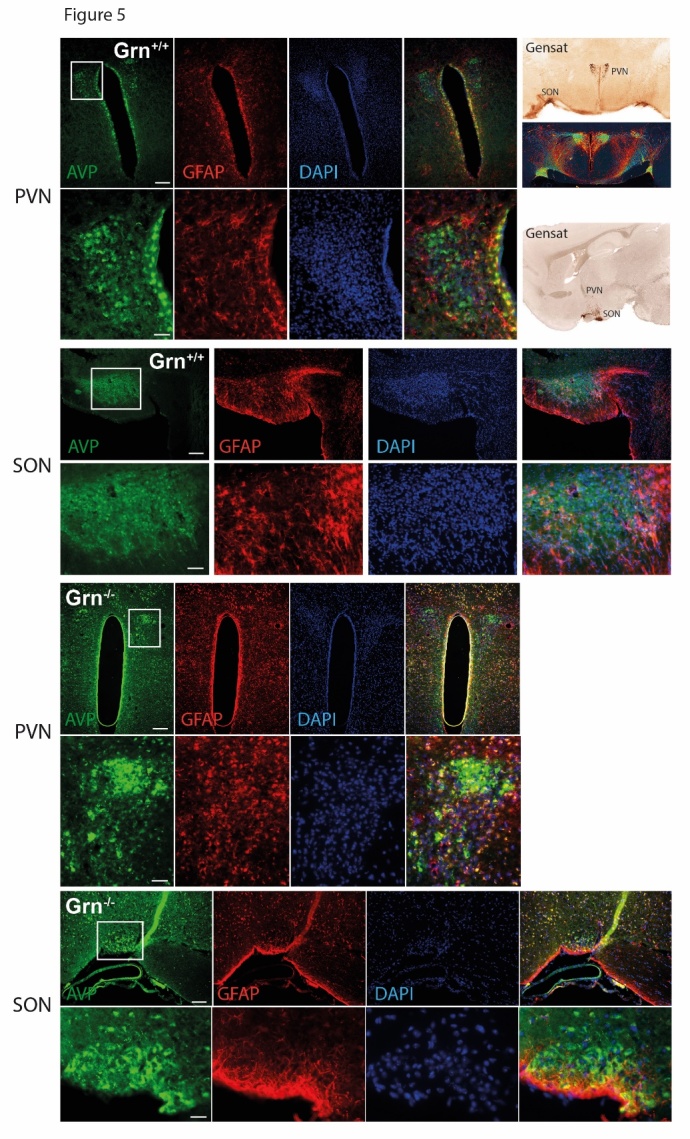
Immunofluorescence analysis of arginine vasopressin (AVP), glial fibrillary acidic protein (GFAP) of astrocytes and DAPI counterstain of nuclei in the hypothalamus of aged progranulin deficient (Grn^-/-^) and control mice (Grn^+/+^) The Gensat images in the right give an overview of the localization of the paraventricular nucleus (PVN) and the nucleus supraopticus (SON), which are the major sites for vasopressin producing neurons. The upper panels show AVP, GFAP and DAPI in the PVN as overview and zoom-in images, and the lower panels show the SON. AVP immunofluorescence was more intense in Grn^-/-^ and AVP positive neurons appear to be enlarged. GFAP staining reveals astrogliosis in Grn^-/-^. Scale bars 300 µm for overviews and 100 µm for zoom-in images.

**Table 1:** Serum and urine electrolytes in progranulin knockout (Grn^-/-^) and wildtype (Grn^+/+^) female mice.

Genotype	Wks	ml/24h	g	Urine (mmol/l)							Serum (mmol/l)								
		
	Age	Drink	BW	Crea	Ca	Cl	K	Mg	Na	P	Glc	Urea	Ca	Cl	K	Mg	Na	P	mosmol/kg
Grn-/-	42.3	7.5	25.5	1.768	4.1	50.6	105.7	3.4	37	11.7	13.1	10	2.3	111	4.3	0.9	146	2.0	315.1
Grn-/-	42.3	9.0	26.6	1.800	3.4	55.1	96.2	3.5	42	12.5	14.5	9.6	2.3	111	4.9	1.1	145	2.0	314.1
Grn-/-	42.3	3.3	25.2	1.774	2.6	29.1	74.9	0.8	31	10.1	12.7	9.7	2.3	112	4.9	1.0	149	1.8	320.4
Grn-/-	42.3	3.8	25.4	1.298	3.2	41.9	85.4	3.0	40	12.4	14.0	8.9	2.3	108	3.8	1.0	143	2.0	308.9
Grn-/-	42.3	2.7	26.0	1.544	5.1	55.9	87.8	3.3	61	2.0	13.0	9.2	2.4	110	4.1	1.1	147	2.3	316.2
Grn-/-	46.4	8.7	24.8	0.413	0.4	17.2	28.2	0.6	16	9.2	16.3	7.3	2.3	112	4.1	0.9	146	2.0	315.6
Grn-/-	51.1	6.0	27.2	1.091	2.1	45.2	74.6	0.3	41	10.9	15.6	7.2	2.4	114	4.0	0.9	149	2.0	320.8

Grn+/+	50.7	2.6	24.0	3.400	6.3	38.9	154.2	3.4	38	14.4	14.9	7.1	2.4	111	3.7	1.0	147	2.0	316.0
Grn+/+	48.6	1.1	23.7	2.831	3.2	37.2	120.2	3.4	45	14.7	13.1	10.2	2.4	112	4.2	1.0	150	2.0	323.3
Grn+/+	48.6	1.2	24.2	1.475	4.3	57.8	114.9	3.4	56	13.8	8.8	12.1	2.4	112	5.2	0.9	148	1.4	316.9
Grn+/+	44.7	0.8	24.0	1.581	5.6	40.4	98.0	3.4	47	6.1	11.4	6.8	2.3	112	4.3	0.9	149	1.6	316.2
Grn+/+	43.6	0.3	23.3	2.288	2.6	48.2	215.9	3.5	35	9.9	13.5	7.4	2.4	109	4.3	0.9	146	1.8	312.9
Grn+/+	43.7	4.7	24.1	1.704	3.5	39.6	88.1	3.3	28	13.3	-	8.8	2.4	112	3.9	1.0	148	2.0	-
Means																			
Grn-/-	44.1	5.9	25.8	1.384	3.0	42.1	78.9	2.1	38.3	9.8	14.2	8.8	2.3	111.1	4.3	1.0	146.4	2.0	315.9
Grn+/+	46.6	1.8	23.9	2.213	4.2	43.7	131.9	3.4	41.5	12.0	12.3	8.7	2.4	111.3	4.3	1.0	148.0	1.8	317.1

Grn-/-SD	3.5	2.6	0.8	0.506	1.5	14.3	25.0	1.5	13.5	3.6	1.4	1.1	0.0	1.9	0.4	0.1	2.1	0.1	4.0
Grn+/+SD	3.0	1.6	0.3	0.775	1.4	7.9	47.0	0.1	9.9	3.4	2.3	2.1	0.0	1.2	0.5	0.1	1.4	0.3	3.8
t-test P	0.196	0.007	0.0003	0.041	0.154	0.820	0.025	0.057	0.640	0.290	0.118	0.907	0.053	0.835	0.902	0.416	0.155	0.083	0.6178

Abbreviations: Wks, weeks; BW, body weight; Crea, creatinine; Ca, calcium; Cl, chloride; K, potassium; Mg, magnesium; Na, sodium; P, anorganic phosphate, Glc, glucose; SD, standard deviation; t-test 2 sided unpaired

### Vasopressin neurons in the hypothalamus

High vasopressin plasma levels in combination with polyuria suggested a counter-regulatory increase of vasopression production in the hypothalamus. In addition, the hypothalamus may be target of the neurodegenerative disease caused by progranulin deficiency. Therefore, we assessed putative signs of hypothalamic gliosis, and number and distribution of vasopression-positive neurons (Fig. 5). Vasopressin (AVP; arginine vasopressin) immunofluorescence was stronger in progranulin deficient brains in the paraventricular (PVN) and supraoptical nuclei (SON), and GFAP immunoreactivity of astrocytes revealed a strong gliosis within these regions in Grn^-/-^ mice, which is in line with the inherent neurodegenerative disease. In controls, AVP staining was finely distributed to fibers, particularly, in SON. In Grn^-/^ mice^-^, AVP-positive neurons appeared to be enlarged but rather reduced in numbers as revealed by DAPI counterstaining of the nuclei.

## DISCUSSION

We show that aged progranulin deficient mice eat and particularly, drink considerable more than controls, about twice the normal volume. Considering that progranulin knockout is a model of frontotemporal dementia, which is predominated in humans by behavioral abnormalities including compulsive eating and drinking, one may hypothesize that the observed phenotype is a manifestation of the frontotemporal neurodegeneration, possibly contributed by dysfunctions of hypothalamic neurons that show comparably high expression of progranulin [41]. Indeed, progranulin’s plasma levels fluctuate with the feeding conditions [27], and the observed inverse dysregulations of ghrelin and agouti related protein in the knockouts point to losses of progranulin-mediated anti-orexigenic effects [27] that may explain overfeeding.

Hence, polydipsia and polyuria could be signs of psychogenic primary polydipsia or central diabetes insipidus due to vasopressin deficiency or mal-processing [42], but both was not the case. Instead, vasopressin levels were increased, and polyuria maintained during water restriction. Except for the water loss, renal functions of progranulin deficient mice were in the normal range agreeing with previous studies [6, 34], and histo-morphometric readouts of chronic renal diseases were negative. There was also no sign of osmotic diuresis due to excretion of glucose. Hence, the results point to a resistance of collecting tubules towards vasopressin resulting in a loss of aquaporin-2 expression and adaptive hypertrophy to handle the increased rates of glomerular filtration and tubular reabsorption that occur in models of central or nephrogenic diabetes insipidus [38, 43].

Vasopressin enhances water reabsorption in the renal collecting duct by vasopressin V2 receptor-mediated activation of adenylylcyclase (AC) [44], cAMP-mediated phosphorylation of aquaporin-2 (AQP2), and increased insertion of AQP2 into the apical membrane [45]. Vasopressin also determines aquaporin-2 transcription via cAMP response elements [46]. In mouse models of nephrogenic diabetes insipidus, collecting ducts are resistant towards vasopressin owing to V2 mutations [47], exaggerated phosphodiesterase-mediated AC degradation [48], interference with AC activity [49], or AQP2 deficiency or malfunction [43, 50, 51]. In particular, age-associated nephrogenic diabetes insipidus in some inbred mouse strains [52, 53] and WAG/Rij rats [54] are caused by an age-progressive decline of AQP2 suggesting that progranulin deficiency may lead to similar but premature AQP2 losses and hence, early manifestations of kidney aging. Considering progranulin’s multi-faceted functions a number of mechanisms may contribute to the DI phenotype.

The observed renal immune cell infiltration in Grn^-/-^ kidney may result from a general pro-inflammatory state in consequence of the loss of immune silencing provided by progranulin, which is expressed in immune cells of myeloid origin and suppressor T-cells and promotes the clearance of pathogens [34, 55]. Permanent immune over-activation in the absence of progranulin may not only affect the kidney, but possibly also lung, liver and brain [34] and may contribute to the observed liver enlargement, possibly a sign of liver steatosis. Hence, although the immune infiltrates do not explain the water loss they may be representatives of the overall pro-inflammatory state.

Intracellular progranulin is localized to "membrane-bounded organelles" which are vesicles of altering destination, including endosomes, autophagosomes and lysosomes. Gene ontology analyses suggest that progranulin is involved in vesicle transport and vesicular transmembrane transport of ions and proteins [23]. Hence, its deficiency may impair vesicular uptake and release of ions and transport of water channels to and from the apical membrane of the collecting tubules. In line with this idea, gene regulation studies in postmortem brain of PGRN-associated FTLD-U patients show deregulations of aquaporin-1 and AQP4 among the top 100 deregulated genes (Geo data sets GDS3459, GSE13162 [56]), raising the intriguing possibility that patients with PGRN-associated frontotemporal lobar degeneration may eventually develop symptoms of polyuria. However so far, diabetes insipidus has not been described as clinical problem in these patients, possibly owing to the early onset and fatal course of the disease and/or interpretation of subtle manifestations as psychogenic polydipsia.

Secreted progranulin binds to and activates a number receptors including epidermal growth factor receptor [57], Ephrin A2 [13] and Notch receptors [12]. Ephrin and Notch signaling is mainly recognized for their pro-fibrotic effects in the kidney, hence not explaining the progranulin-provided benefit, but inactivation of Notch signaling in the renal collecting duct causes nephrogenic diabetes insipidus [58] similar to that seen in our progranulin-deficient mice. Similar phenotypes of nephrogenic DI were observed in aldose reductase deficient mice [51] and calcineurin deficient mice [59] all converging on losses of AQP2 expression, transport or phosphorylation, however without known interconnection with progranulin.

The increase of vasopressin plasma levels likely reflects an intact adaptation of the hypothalamic-hypophyseal axis in response to the renal water loss and argues against a substantial degeneration of hormone producing neurons at that age. However, AVP positive neurons appear to be enlarged and are surrounded by astrogliosis suggesting that the hypothalamus is affected by progranulin deficiency. In line, the observed changes of hormones regulating appetite and feeding show that progranulin is involved in hormone homeostasis. Hypothalamic dysfunctions have been observed in some other mouse models of neurodegenerative diseases [60, 61], and primary polydipsia was found in saposin D deficient mice [26], the latter important because its precursor, prosaposin and progranulin converge on sortilin-mediated transport, interaction at the autophagolysosomal interface [16] and lysosomal functions [18, 62]. In contrast to our mice, saposin D deficient mice had reduced vasopressin levels, and water restriction resulted in a normalization of kidney morphology and functions [26], suggesting different mechanisms of the polydipsia-polyuria phenotype.

In summary, we show that progranulin deficient mice develop a nephrogenic diabetes insipidus, which becomes manifest at about 6-9 month of age with a cumulative incidence of about 70-80%. Polyuria and polydipsia lead to a doubling of drinking volume and urine output but the dysfunction does not lead to extreme water losses, kidney damage or premature death. Nevertheless, the data show a relevant function of progranulin for the maintenance of water homeostasis and osmolality.

## References

[1] Cruts M, Gijselinck I, van der Zee J, Engelborghs S, Wils H, Pirici D, Rademakers R, et al (2006). Null mutations in progranulin cause ubiquitin-positive frontotemporal dementia linked to chromosome 17q21. Nature, 442: 920-4. 16862115 10.1038/nature05017

[2] Baker M, Mackenzie IR, Pickering-Brown SM, Gass J, Rademakers R, Lindholm C, et al (2006). Mutations in progranulin cause tau-negative frontotemporal dementia linked to chromosome 17. Nature, 442: 916-9. 16862116 10.1038/nature05016

[3] Gotzl JK, Mori K, Damme M, Fellerer K, Tahirovic S, Kleinberger G, et al (2014). Common pathobiochemical hallmarks of progranulin-associated frontotemporal lobar degeneration and neuronal ceroid lipofuscinosis. Acta Neuropathol, 127: 845-60. 24619111 10.1007/s00401-014-1262-6

[4] Mackenzie IR, Baker M, Pickering-Brown S, Hsiung GY, Lindholm C, Dwosh E, Gass J, Cannon A, Rademakers R, Hutton M, Feldman HH (2006). The neuropathology of frontotemporal lobar degeneration caused by mutations in the progranulin gene. Brain, 129: 3081-90. 17071926 10.1093/brain/awl271

[5] Hardt S, Heidler J, Albuquerque B, Valek L, Altmann C, Wilken-Schmitz A, Schafer MKE, Wittig I, Tegeder I (2017). Loss of synaptic zinc transport in progranulin deficient mice may contribute to progranulin-associated psychopathology and chronic pain. Biochim Biophys Acta. 1863:2727-2745 10.1016/j.bbadis.2017.07.014 28720486

[6] Yin F, Dumont M, Banerjee R, Ma Y, Li H, Lin MT, Beal MF, Nathan C, Thomas B, Ding A (2010). Behavioral deficits and progressive neuropathology in progranulin-deficient mice: a mouse model of frontotemporal dementia. FASEB J, 24: 4639-47. 20667979 10.1096/fj.10-161471 PMC2992364

[7] Ahmed Z, Sheng H, Xu YF, Lin WL, Innes AE, Gass J, et al (2010). Accelerated lipofuscinosis and ubiquitination in granulin knockout mice suggest a role for progranulin in successful aging. Am J Pathol, 177: 311-24. 20522652 10.2353/ajpath.2010.090915 PMC2893674

[8] Albuquerque B, Haussler A, Vannoni E, Wolfer DP, Tegeder I (2013). Learning and memory with neuropathic pain: impact of old age and progranulin deficiency. Front Behav Neurosci, 7: 174. 24319417 10.3389/fnbeh.2013.00174 PMC3837228

[9] Filiano AJ, Martens LH, Young AH, Warmus BA, Zhou P, Diaz-Ramirez G, Jiao J, Zhang Z, Huang EJ, Gao FB, Farese RV Jr., Roberson ED (2013). Dissociation of frontotemporal dementia-related deficits and neuroinflammation in progranulin haploinsufficient mice. J Neurosci, 33: 5352-61. 23516300 10.1523/JNEUROSCI.6103-11.2013 PMC3740510

[10] Hu F, Padukkavidana T, Vaegter CB, Brady OA, Zheng Y, Mackenzie IR, Feldman HH, Nykjaer A, Strittmatter SM (2010). Sortilin-mediated endocytosis determines levels of the frontotemporal dementia protein, progranulin. Neuron, 68: 654-67. 21092856 10.1016/j.neuron.2010.09.034 PMC2990962

[11] He Z, Bateman A (1999). Progranulin gene expression regulates epithelial cell growth and promotes tumor growth in vivo. Cancer Res, 59: 3222-9. 10397269

[12] Altmann C, Vasic V, Hardt S, Heidler J, Haussler A, Wittig I, Schmidt MH, Tegeder I (2016). Progranulin promotes peripheral nerve regeneration and reinnervation: role of notch signaling. Mol neurodegener, 11: 69. 27770818 10.1186/s13024-016-0132-1 PMC5075406

[13] Neill T, Buraschi S, Goyal A, Sharpe C, Natkanski E, Schaefer L, Morrione A, Iozzo RV (2016). EphA2 is a functional receptor for the growth factor progranulin. J Cell Biol, 215: 687-703. 27903606 10.1083/jcb.201603079 PMC5146997

[14] Gass J, Lee WC, Cook C, Finch N, Stetler C, Jansen-West K, Lewis J, Link CD, Rademakers R, Nykjaer A, Petrucelli L (2012). Progranulin regulates neuronal outgrowth independent of Sortilin. Mol Neurodegener, 7: 33. 22781549 10.1186/1750-1326-7-33 PMC3508877

[15] Sikora J, Harzer K, Elleder M (2007). Neurolysosomal pathology in human prosaposin deficiency suggests essential neurotrophic function of prosaposin. Acta Neuropathol, 113: 163-75. 17024494 10.1007/s00401-006-0148-7 PMC2956888

[16] Zhou X, Sun L, Bastos de Oliveira F, Qi X, Brown WJ, Smolka MB, Sun Y, Hu F (2015). Prosaposin facilitates sortilin-independent lysosomal trafficking of progranulin. J Cell Biol, 210: 991-1002. 26370502 10.1083/jcb.201502029 PMC4576858

[17] Matsuda J, Kido M, Tadano-Aritomi K, Ishizuka I, Tominaga K, Toida K, Takeda E, Suzuki K, Kuroda Y (2004). Mutation in saposin D domain of sphingolipid activator protein gene causes urinary system defects and cerebellar Purkinje cell degeneration with accumulation of hydroxy fatty acid-containing ceramide in mouse. Hum Mol Genet, 13: 2709-23. 15345707 10.1093/hmg/ddh281

[18] Sun Y, Witte DP, Zamzow M, Ran H, Quinn B, Matsuda J, Grabowski GA (2007). Combined saposin C and D deficiencies in mice lead to a neuronopathic phenotype, glucosylceramide and alpha-hydroxy ceramide accumulation, and altered prosaposin trafficking. Hum Mol Genet, 16: 957-71. 17353235 10.1093/hmg/ddm040

[19] Matsuda J, Yoneshige A, Suzuki K (2007). The function of sphingolipids in the nervous system: lessons learnt from mouse models of specific sphingolipid activator protein deficiencies. J Neurochem, 103 Suppl 1: 32-8. 17986137 10.1111/j.1471-4159.2007.04709.x

[20] He Z, Ong CH, Halper J, Bateman A (2003). Progranulin is a mediator of the wound response. Nat Med, 9: 225-9. 12524533 10.1038/nm816

[21] Tang W, Lu Y, Tian QY, Zhang Y, Guo FJ, Liu GY, et al (2011). The growth factor progranulin binds to TNF receptors and is therapeutic against inflammatory arthritis in mice. Science, 332: 478-84. 21393509 10.1126/science.1199214 PMC3104397

[22] Zhou M, Tang W, Fu Y, Xu X, Wang Z, Lu Y, Liu F, Yang X, Wei X, Zhang Y, Liu J, Geng X, Zhang C, Wan Q, Li N, Yi F (2015). Progranulin protects against renal ischemia/reperfusion injury in mice Kidney Int, 87: 918-29. 10.1038/ki.2014.403 25607110

[23] Altmann C, Hardt S, Fischer C, Heidler J, Lim HY, Haussler A, et al (2016). Progranulin overexpression in sensory neurons attenuates neuropathic pain in mice: Role of autophagy. Neurobiol Dis, 96: 294-311. 27629805 10.1016/j.nbd.2016.09.010

[24] Tanaka Y, Chambers JK, Matsuwaki T, Yamanouchi K, Nishihara M (2014). Possible involvement of lysosomal dysfunction in pathological changes of the brain in aged progranulin-deficient mice. Acta neuropathol commun, 2: 78. 25022663 10.1186/s40478-014-0078-x PMC4149276

[25] Perez Y, Shorer Z, Liani-Leibson K, Chabosseau P, Kadir R, Volodarsky M, Halperin D, Barber-Zucker S, Shalev H, Schreiber R, Gradstein L, Gurevich E, Zarivach R, Rutter GA, Landau D, Birk OS (2017). SLC30A9 mutation affecting intracellular zinc homeostasis causes a novel cerebro-renal syndrome. Brain, 140: 928-939. 28334855 10.1093/brain/awx013 PMC5837213

[26] Hisaki H, Matsuda J, Tadano-Aritomi K, Uchida S, Okinaga H, Miyagawa M, Tamamori-Adachi M, Iizuka M, Okazaki T (2012). Primary polydipsia, but not accumulated ceramide, causes lethal renal damage in saposin D-deficient mice. Am J Physiol Renal Physiol, 303: F1049-59. 22832923 10.1152/ajprenal.00047.2012

[27] Kim HK, Shin MS, Youn BS, Namkoong C, Gil SY, Kang GM, Yu JH, Kim MS (2011). Involvement of progranulin in hypothalamic glucose sensing and feeding regulation. Endocrinology, 152: 4672-82. 21933869 10.1210/en.2011-1221

[28] Suzuki M, Nishiahara M (2002). Granulin precursor gene: a sex steroid-inducible gene involved in sexual differentiation of the rat brain. Mol Genet Metab, 75: 31-7. 11825061 10.1006/mgme.2001.3274

[29] Zhou B, Li H, Liu J, Xu L, Guo Q, Sun H, Wu S (2015). Progranulin induces adipose insulin resistance and autophagic imbalance via TNFR1 in mice. J Mol Endocrinol, 55: 231-43. 26373796 10.1530/JME-15-0075

[30] Nguyen AD, Nguyen TA, Martens LH, Mitic LL, Farese RV Jr., (2013). Progranulin: at the interface of neurodegenerative and metabolic diseases. Trends Endocrinol Metab, 24: 597-606. 24035620 10.1016/j.tem.2013.08.003 PMC3842380

[31] Tegeder I (2016). Yeast-2-Hybrid data file showing progranulin interactions in human fetal brain and bone marrow libraries. Data Brief, 9: 1060-1062. 27921076 10.1016/j.dib.2016.11.031 PMC5126125

[32] Nedachi T, Kawai T, Matsuwaki T, Yamanouchi K, Nishihara M (2011). Progranulin enhances neural progenitor cell proliferation through glycogen synthase kinase 3beta phosphorylation. Neurosci, 185: 106-15. 10.1016/j.neuroscience.2011.04.037 21540081

[33] Norregaard R, Tao S, Nilsson L, Woodgett JR, Kakade V, Yu AS, Howard C, Rao R (2015). Glycogen synthase kinase 3alpha regulates urine concentrating mechanism in mice. Am J Physiol Renal Physiol, 308: F650-60. 25608967 10.1152/ajprenal.00516.2014 PMC4360031

[34] Yin F, Banerjee R, Thomas B, Zhou P, Qian L, Jia T, Ma X, Ma Y, Iadecola C, Beal MF, Nathan C, Ding A (2010). Exaggerated inflammation, impaired host defense, and neuropathology in progranulin-deficient mice. J Exp Med, 207: 117-28. 20026663 10.1084/jem.20091568 PMC2812536

[35] Voikar V, Colacicco G, Gruber O, Vannoni E, Lipp HP, Wolfer DP (2010). Conditioned response suppression in the IntelliCage: assessment of mouse strain differences and effects of hippocampal and striatal lesions on acquisition and retention of memory. Behav Brain Res, 213: 304-12. 20493907 10.1016/j.bbr.2010.05.019

[36] Krackow S, Vannoni E, Codita A, Mohammed AH, Cirulli F, Branchi I, Alleva E, Reichelt A, Willuweit A, Voikar V, Colacicco G, Wolfer DP, Buschmann JU, Safi K, Lipp HP (2010). Consistent behavioral phenotype differences between inbred mouse strains in the IntelliCage. Genes Brain Behav, 9: 722-31. 20528956 10.1111/j.1601-183X.2010.00606.x

[37] Moreth K, Frey H, Hubo M, Zeng-Brouwers J, Nastase MV, Hsieh LT, Haceni R, Pfeilschifter J, Iozzo RV, Schaefer L (2014). Biglycan-triggered TLR-2- and TLR-4-signaling exacerbates the pathophysiology of ischemic acute kidney injury. Matrix Biology, 35: 143-51. 24480070 10.1016/j.matbio.2014.01.010 PMC4057019

[38] Naccarato R, Rizzo A, Sirigu F, Bertaglia E, Previato G, Fiaschi E (1976). Renal histologic and ultrastructural findings in psychogenic polydipsia and diabetes insipidus. Nephron, 16: 226-35. 1244580 10.1159/000180606

[39] Schliebe N, Strotmann R, Busse K, Mitschke D, Biebermann H, Schomburg L, et al (2008). V2 vasopressin receptor deficiency causes changes in expression and function of renal and hypothalamic components involved in electrolyte and water homeostasis. Am J Physiol Renal Physiol, 295: F1177-90. 18715941 10.1152/ajprenal.00465.2007

[40] Sohara E, Rai T, Yang SS, Uchida K, Nitta K, Horita S, Ohno M, Harada A, Sasaki S, Uchida S (2006). Pathogenesis and treatment of autosomal-dominant nephrogenic diabetes insipidus caused by an aquaporin 2 mutation. Proc Natl Acad Sci U S A, 103: 14217-22. 16968783 10.1073/pnas.0602331103 PMC1599937

[41] Matsuwaki T, Asakura R, Suzuki M, Yamanouchi K, Nishihara M (2011). Age-dependent changes in progranulin expression in the mouse brain. J Reprod Dev, 57: 113-9. 20962456 10.1262/jrd.10-116s

[42] Shi G, Somlo D, Kim GH, Prescianotto-Baschong C, Sun S, Beuret N, Long Q, Rutishauser J, Arvan P, Spiess M, Qi L (2017). ER-associated degradation is required for vasopressin prohormone processing and systemic water homeostasis. J Clin Invest. 127(10): 3897-3912 10.1172/JCI94771 PMC5617659 28920920

[43] Yang B, Zhao D, Qian L, Verkman AS (2006). Mouse model of inducible nephrogenic diabetes insipidus produced by floxed aquaporin-2 gene deletion. Am J Physiol Renal Physiol, 291: F465-72. 16434568 10.1152/ajprenal.00494.2005

[44] Poulsen SB, Kristensen TB, Brooks HL, Kohan DE, Rieg T, Fenton RA (2017). Role of adenylyl cyclase 6 in the development of lithium-induced nephrogenic diabetes insipidus. JCI insight, 2: e91042. 28405619 10.1172/jci.insight.91042 PMC5374078

[45] Cano-Penalver JL, Griera M, Serrano I, Rodriguez-Puyol D, Dedhar S, de Frutos S, Rodriguez-Puyol M (2014). Integrin-linked kinase regulates tubular aquaporin-2 content and intracellular location: a link between the extracellular matrix and water reabsorption. FASEB J, 28: 3645-59. 24784577 10.1096/fj.13-249250

[46] Hozawa S, Holtzman EJ, Ausiello DA (1996). cAMP motifs regulating transcription in the aquaporin 2 gene Am J Physiol, 270: C1695-702. 8764152 10.1152/ajpcell.1996.270.6.C1695

[47] Birnbaumer M, Gilbert S, Rosenthal W (1994). An extracellular congenital nephrogenic diabetes insipidus mutation of the vasopressin receptor reduces cell surface expression, affinity for ligand, and coupling to the Gs/adenylyl cyclase system. Mol Endocrinol, 8: 886-94. 7984150 10.1210/mend.8.7.7984150

[48] Valtin H, Coffey AK, O’Sullivan DJ, Homma S, Dousa TP (1990). Causes of the urinary concentrating defect in mice with nephrogenic diabetes insipidus. Physiologia Bohemoslovaca, 39: 103-11. 2165265

[49] Rao R, Patel S, Hao C, Woodgett J, Harris R (2010). GSK3beta mediates renal response to vasopressin by modulating adenylate cyclase activity. J Am Soc Nephrol, 21: 428-37. 20056751 10.1681/ASN.2009060672 PMC2831860

[50] Kortenoeven ML, Pedersen NB, Miller RL, Rojek A, Fenton RA (2013). Genetic ablation of aquaporin-2 in the mouse connecting tubules results in defective renal water handling. J Physiol, 591: 2205-19. 23359673 10.1113/jphysiol.2012.250852 PMC3634529

[51] Ho HT, Chung SK, Law JW, Ko BC, Tam SC, Brooks HL, Knepper MA, Chung SS (2000). Aldose reductase-deficient mice develop nephrogenic diabetes insipidus. Mol Cell Biol, 20: 5840-6. 10913167 10.1128/mcb.20.16.5840-5846.2000 PMC86061

[52] Tsumura K, Li X, Murdiastuti K, Parvin MN, Akamatsu T, Yao C, Kanamori N, Inenaga K, Yamashita H, Hosoi K (2006). Downregulation of AQP2 expression in the kidney of polydipsic STR/N mice. Am J Physiol Renal Physiol, 290: F478-85. 16144968 10.1152/ajprenal.00029.2005

[53] Kutscher CL, Miller M, Schmalbach NL (1975). Renal deficiency associated with diabetes insipidus in the SWR/J mouse. Physiol Behav, 14: 815-8. 1187837 10.1016/0031-9384(75)90075-x

[54] Preisser L, Teillet L, Aliotti S, Gobin R, Berthonaud V, Chevalier J, Corman B, Verbavatz JM (2000). Downregulation of aquaporin-2 and -3 in aging kidney is independent of V(2) vasopressin receptor. Am J Physiol Renal Physiol, 279: F144-52. 10894796 10.1152/ajprenal.2000.279.1.F144

[55] Hoque M, Young TM, Lee CG, Serrero G, Mathews MB, Pe’ery T (2003). The growth factor granulin interacts with cyclin T1 and modulates P-TEFb-dependent transcription. Mol Cell Biol, 23: 1688-702. 12588988 10.1128/MCB.23.5.1688-1702.2003 PMC151712

[56] Chen-Plotkin AS, Geser F, Plotkin JB, Clark CM, Kwong LK, Yuan W, Grossman M, Van Deerlin VM, Trojanowski JQ, Lee VM (2008). Variations in the progranulin gene affect global gene expression in frontotemporal lobar degeneration. Hum Mol Genet, 17: 1349-62. 18223198 10.1093/hmg/ddn023 PMC2900863

[57] Bateman A, Bennett HP (1998). Granulins: the structure and function of an emerging family of growth factors. J Endocrinol, 158: 145-51. 9771457 10.1677/joe.0.1580145

[58] Jeong HW, Jeon US, Koo BK, Kim WY, Im SK, Shin J, Cho Y, Kim J, Kong YY (2009). Inactivation of Notch signaling in the renal collecting duct causes nephrogenic diabetes insipidus in mice. J Clin Invest, 119: 3290-300. 19855135 10.1172/JCI38416 PMC2769200

[59] Gooch JL, Guler RL, Barnes JL, Toro JJ (2006). Loss of calcineurin Aalpha results in altered trafficking of AQP2 and in nephrogenic diabetes insipidus. J Cell Sci, 119: 2468-76. 16735444 10.1242/jcs.02971

[60] Kotliarova S, Jana NR, Sakamoto N, Kurosawa M, Miyazaki H, Nekooki M, Doi H, Machida Y, Wong HK, Suzuki T, Uchikawa C, Kotliarov Y, Uchida K, Nagao Y, Nagaoka U, Tamaoka A, Oyanagi K, Oyama F, Nukina N (2005). Decreased expression of hypothalamic neuropeptides in Huntington disease transgenic mice with expanded polyglutamine-EGFP fluorescent aggregates. J Neurochem, 93: 641-53. 15836623 10.1111/j.1471-4159.2005.03035.x

[61] Wood NI, Goodman AO, van der Burg JM, Gazeau V, Brundin P, Bjorkqvist M, Petersen A, Tabrizi SJ, Barker RA, Morton AJ (2008). Increased thirst and drinking in Huntington’s disease and the R6/2 mouse. Brain Res Bull, 76: 70-9. 18395613 10.1016/j.brainresbull.2007.12.007

[62] Tanaka Y, Suzuki G, Matsuwaki T, Hosokawa M, Serrano G, Beach TG, Yamanouchi K, Hasegawa M, Nishihara M (2017). Progranulin regulates lysosomal function and biogenesis through acidification of lysosomes. Hum Mol Genet, 26(5):969-988 28073925 10.1093/hmg/ddx011

